# A Model-Driven Approach to Assessing the Fouling Mechanism in the Crossflow Filtration of Laccase Extract from *Pleurotus ostreatus* 202

**DOI:** 10.3390/membranes15080226

**Published:** 2025-07-29

**Authors:** María Augusta Páez, Mary Casa-Villegas, Vanesa Naranjo-Moreno, Neyda Espín Félix, Katty Cabezas-Terán, Alfonsina Andreatta

**Affiliations:** 1 Departamento de Ciencia de Alimentos y Biotecnología, Facultad de Ingeniería Química y Agroindustria, Escuela Politécnica Nacional, Ladrón de Guevara E11-253, Quito 170143, Ecuador; mary.casa@epn.edu.ec (M.C.-V.); samanta.naranjo@epn.edu.ec (V.N.-M.); neyda.espin@epn.edu.ec (N.E.F.); katty.cabezas@epn.edu.ec (K.C.-T.); 2 Ingeniería de Procesos Sustentables (InProSus)–Consejo Nacional de Investigaciones Científicas y Técnicas (CONICET), Facultad Regional San Francisco, Universidad Tecnológica Nacional, Av. de la Universidad 501, San Francisco CP 2400, Córdoba, Argentina; aandreatta@facultad.sanfrancisco.utn.edu.ar

**Keywords:** fouling, crossflow filtration, pore blocking, permeate flux, filtration models, laccases

## Abstract

Membrane technology is primarily used for the separation and purification of biotechnological products, which contain proteins and enzymes. Membrane fouling during crossflow filtration remains a significant challenge. This study aims to initially validate crossflow filtration models, particularly related to pore-blocking mechanisms, through a comparative analysis with dead-end filtration models. One crossflow microfiltration (MF) and six consecutive ultrafiltration (UF) stages were implemented to concentrate laccase extracts from *Pleurotus ostreatus* 202 fungi. The complete pore-blocking mechanism significantly impacts the MF, UF 1000, UF 100 and UF 10 stages, with the highest related filtration constant (Kb_F_) estimated at 12.60 × 10^−4^ (m^−1^). Although the intermediate pore-blocking mechanism appears across all filtration stages, UF 100 is the most affected, with an associated filtration constant (Ki_F_) of 16.70 (m^−1^). This trend is supported by the highest purification factor (6.95) and the presence of 65, 62 and 56 kDa laccases in the retentate. Standard pore blocking occurs at the end of filtration, only in the MF and UF 1000 stages, with filtration constants (Ks_F_) of 29.83 (s^−0.5^m^−0.5^) and 31.17 (s^−0.5^m^−0.5^), respectively. The absence of cake formation and the volume of permeate recovered indicate that neither membrane was exposed to exhaustive fouling that could not be reversed by backwashing.

## 1. Introduction

Tangential flow filtration, also referred to as crossflow filtration, is a downstream process based on the action of pressure to purify solutes and enhance filtrate quality by means of selective filtration in a membrane [1]. Unlike dead-end filtration, the flow direction is parallel to the membrane surface and a relatively high flow rate prevents concentration polarization [2]. Transmembrane pressure drives fluid flow through the membrane, while crossflow velocity generates a force that sweeps molecules away from the membrane surface, thereby promoting back-diffusion and minimizing flux decline [3]. Membrane technology is widely used in the separation and purification of biological products [3,4]. Microfiltration (MF) is commonly used to remove cell debris, particles (0.1–10 microns) and bacteria from protein-containing feeds [2,3]. Membrane separation with proteins is an important part of bioprocessing applications, as it is an evolving trend to separate proteins while preserving activity and structure and achieving higher resolution [1]. In this field, ultrafiltration (UF) is mainly used for the concentration of proteins, but it is limited to isolate proteins of relatively close molecular weight [2,3]. Bovine serum albumin (BSA) is the most frequently used protein, but ovalbumin, pepsin, casein and hemoglobin have also been found [2]. In addition to proteins, UF studies also encompass enzymes such as lysozymes [5,6], laccases [7,8,9] and proteases [10,11]. Laccases are copper-containing biocatalysts that exhibit superior stability and resistance to organic solvents [12]. These enzymes possess the potential ability to oxidize both phenolic and non-phenolic compounds, and their molecular weight ranges from 50 to 130 kDa [12,13]. Laccases are secreted during the secondary metabolism of wood-decaying fungi, including *Phanerochaete chrysosporium*, *Trametes versicolor*, *Cerena maxima*, *Coriolposis polyzona* and *Pleurotus ostreatus*, *Ganoderma lucidum*, *Cerrena unicolor* and *Coriolopsis gallica* [12,14]. Among them, *P. ostreatus* is regarded as a model laccase-producing strain with remarkable yield, especially in solid culture [14,15]. The considerable versatility of laccases is evident in their extensive range of industrial applications, encompassing sectors such as paper and pulp production, textile industry, bioremediation and food processing [12,13]. Researchers are currently working to enhance efficiency and productivity by identifying suitable microorganisms and optimizing the production and purification processes [13].

When it comes to proteins and enzymes, including laccases, the primary challenge in implementing effective MF and UF is the rate of fouling, which is significantly influenced by the concentration and characteristics of the incoming feed, as well as the membrane material [4]. Fouling and the subsequent decline in permeate flux occur in three stages: (1) the formation of a reversible concentration polarization boundary layer, (2) the adsorption and deposition of protein on the membrane surface and subsurface (into the pores), and (3) the quasi-stationary convective deposition of proteins [1,2]. According to the theories on membrane fouling, the deposition and adsorption of particles causing pore sealing can be described by intermediate, standard, and complete blocking mechanisms. Cake layer formation, on the other hand, reflects only the deposition of particles that are larger than the pore size [16]. Hermia [17] was the first to provide the physical derivation of the blocking law for dead-end filtration under constant pressure and presented a single equation that links the four blocking mechanisms, as follows:

(1) $$\frac{d^{2}t}{dV^{2}}=K_{n}{\left(\frac{dt}{dV}\right)}^{n}$$

where V is the filtrate volume, n is an index characteristic of a particular blocking mode (*n* = 2 complete pore, *n* = 1.5 standard pore, *n* = 1 intermediate pore and *n* = 0 cake formation), and $K_{n}$ is a phenomenological constant depending upon the blocking mode. The mathematical integral evaluation [18] and the corresponding linearized equation [19] for each fouling mechanism allows for an evaluation based mainly on the fitting parameter R^2^. Under this scheme, the fouling analysis for crossflow UF was performed with polyethylene glycol (PEG) solutions in Carbosep M2 monotubular ceramic membranes [20,21] and BSA solutions in hydrophobic Durapore membranes [22]. To the best of our knowledge, no studies have investigated membrane fouling during the concentration of laccases. Crossflow inhibits the buildup of particles, so the associated mass balances must include a removal term that is independent of flux and probably proportional to shear stress [21]. Field, Wu, Howell and Gupta [18] modified the dead-end model to be applied to crossflow filtration, and later, Field and Wu [23] presented a re-evaluation according to the following expression:

(2) $$-\frac{dJ}{dt}=K_{n}\left(J-J_{R}\right){\;J}^{2-n}$$

where J is the permeate flux, n and $K_{n}$ have the same meaning originally stated and $J_{R}$ can be taken to be the steady-state flux that reflects the removal term. The integration analysis [23] and the five-step methodology for linearization [24] provide the optimal fit to the data and facilitate the identification of the fouling mechanism. According to the critical review by Field and Wu [25], in the development of a robust methodology for the fouling analysis of crossflow systems, the modest increase in the complexity of the models accounting for removal terms should be addressed. Moreover, given the potential for fouling to compromise the technical and economic viability of membrane-based processes, there has been notable advancement in the understanding of the fundamental mechanisms governing the adsorption, desorption and permeation of foulants [6]. This improved understanding becomes a model-based tool for the prediction of filtration performance throughout different stages, enhancing the regulation of filtration flux and fouling mitigation.

This work aims to initially validate crossflow filtration models that describe fouling mechanisms through a comparative analysis with dead-end filtration models. The fouling mechanisms considered include complete pore blocking, intermediate pore blocking, standard blocking, and cake layer formation. The study was conducted in the context of tangential flow treatment, which comprises an initial MF stage, followed by six UF stages, for laccase extracts from *Pleurotus ostreatus* 202.

## 2. Materials and Methods

### 2.1. Microorganisms and Laccase Extract Production

*Pleurotus ostreatus* 202 fungi were provided by Bioprocess Laboratory, Escuela Politécnica Nacional (EPN), Quito, Ecuador, and kept in malt extract agar (MEA) Petri dishes at 30 °C for 14 days.

Laccase extracts were produced as described by Téllez [26] with some modifications. Five mycelial discs (0.5 cm in diameter), obtained from a *P. ostreatus* colony grown on MEA for 7 days, were inoculated into 250 mL flasks containing 1 g of polyurethane foam (PUF) cut into 0.5 cm cubes with a density of 20.55 kg/m^3^, used as an inert support. The PUF was impregnated with 35 mL of culture medium composed of (per liter) 10 g of glucose, 5 g of yeast extract, 0.5 g of MgSO_4_·7H_2_O, 0.05 g of MnSO_4_·H_2_O, 0.4 g of K_2_HPO_4_, 0.6 g of KH_2_PO_4_, 0.25 g of CuSO_4_·5H_2_O, 0.005 g of FeSO_4_·7H_2_O and 0.001 g of ZnSO_4_·7H_2_O. The fermentation was carried out for 12 days at 30 °C in a rotary shaker at 120 rpm. Previously, the PUF cubes were washed twice with distilled water and dried at 60 °C overnight; then, they were mixed with the medium and sterilized at 121 °C for 15 min.

The crude enzyme extract from the 20 flask cultures was obtained by vacuum filtration followed by centrifugation at 10,000 rpm for 20 min, recovering 500 mL as a total volume of extract.

### 2.2. Filtration Experiments

The concentration of laccases from the crude enzyme broth was performed at room temperature in a Labscale™ Tangential Flow Filtration-TFF-System (Millipore, Bedford, MA, USA) consisting of a 500 mL acrylic reservoir with pressure gauges, device docking manifold and retentate valve, system base with integral magnetic stirrer and diaphragm pump [27]. Prior to the filtration experiments, the membranes were properly conditioned by circulating 50 mL of acetate buffer solution at a flow rate corresponding to a feed pressure of 138 kPa. For laccase concentration, conducted at 18 °C, the sample was introduced into the reservoir and circulated by the pump through the filter, maintaining a transmembrane pressure (TMP) of 138 kPa by adjusting the pump speed and the retentate valve. The portion of the sample that passed through the membrane was collected as permeate, while the remainder was the retentate.

Seven different membranes were used sequentially for the concentration of the laccases, such that permeate from each filtration step was used as feed solution for the next process. The crude enzyme broth was first passed through the microfiltration membrane made of polyethersulfone (PES), with a pore diameter of 0.45 µm [27] to remove any suspended material such as cells and debris. The six ultrafiltration membranes (BioMax, Millipore, Bedford, MA, USA) were made of polyethersulfone (PES), with a filtration area of 0.005 m^2^ and molecular weight cutoffs of 1000, 500, 300, 100, 50 and 10 kDa.

After each filtration experiment, the membranes and the TFF system were rinsed with 0.1 N NaOH solution at 40 °C. First, 250 mL of the cleaning solution was pumped at a feed pressure of 138 kPa, with the retentate tubing disconnected and replaced by a waste collection vessel. Then, after reconnecting the retentate tubing, another 250 mL of the cleaning solution was recirculated for 30 to 60 min, depending on the degree of fouling. The pump speed was adjusted to maintain feed and retentate pressures of 209 kPa and 69 kPa, respectively [27]. In each filtration step, the protein level and laccase activity in both permeate and retentate were determined, as detailed below.

### 2.3. Laccase Extract Characterization

#### 2.3.1. Laccase Activity, Protein Concentration and Specific Activity

Laccase activity (Lac) was measured by the 2.2-azino-bis-(3-ethylbenzothiazoline-6-sulfonic acid) (ABTS) oxidation method described by Flores et al. [28] with some modifications. The reaction was conducted with 0.5 mL of 0.5 mM ABTS, 1 mL of 0.1 M sodium acetate buffer solution (pH 4.5) and 0.5 mL of enzymatic extract at 30 °C for 5 min. The absorbance increase was quantified at 420 nm with a spectrometer (Thermo Spectronic GENESYS 20, Thermo Fisher Scientific Inc., Waltham, MA, USA). Laccase activity was expressed in U/mL, where U means 1 µmol of oxidated substrate per minute.

Protein concentration (Pro), expressed in mg/mL, was determined according to the Biuret method originally described by Gornall et al. [29] with some modifications. A volume of 0.5 mL of enzymatic extract was mixed with 2.5 mL of Biuret reagent, which consisted of 0.15% cupric sulfate pentahydrate, 0.6% sodium tartrate dihydrate and 3% sodium hydroxide. Absorbance was measured at 540 nm using a spectrophotometer (Spectro UV-VIS, Labomed Inc., Los Angeles, CA, USA). The absorbance values were interpolated against a calibration curve generated with bovine serum albumin (BSA) to determine the protein concentration.

The specific activity (SA), defined as the amount of enzyme activity per unit of protein in the sample, was calculated as the ratio of laccase activity (Lac) to protein concentration (Pro) and was expressed in U/mg, as follows:

(3) $$SA=\frac{Lac}{Pro}$$

#### 2.3.2. Filtration Performance Parameters

For each filtration step, the volumetric concentration factor (VCF), the activity concentration factor (ACF), recovery efficiency (R) and the purification factor (PF) were calculated according to the equations described by Zaccaria et al. [9] and Antecka et al. [30]. VCF, which indicates the degree to which the feed has been concentrated, was calculated using Equation (4) as the ratio of the initial extract volume ($V_{0}$) to the retentate volume ($V_{r}$).

(4) $$VCF=\frac{V_{0}}{V_{r}}$$

ACF, which indicates the extent to which enzyme activity has been concentrated, was calculated using Equation (5) as the ratio between the laccase activity in the retentate (${Lac}_{r}$) and the laccase activity in the feed (${Lac}_{o}$), both expressed in U/mL. Then, R, which represents the proportion of total enzyme activity recovered in the final product, was calculated using Equation (6) as the ratio between ACF and VCF.

(5) $$ACF=\frac{{Lac}_{r}}{{Lac}_{o}}$$

(6) $$R=\frac{{Lac}_{r}\;V_{r}}{{Lac}_{o}\;V_{o}}$$

The purification factor (PF), which reflects the increase in specific activity following the concentration step, was calculated using Equation (7), dividing the specific activity of the retentate (${SA}_{r}$) by the initial specific activity of the feed (${SA}_{o}$).

(7) $$PF=\frac{{SA}_{r}}{{SA}_{o}}$$

#### 2.3.3. Molecular Weight Determination

In the retentate resulting from the filtration step with the best PF, the molecular weight of the proteins was determined using the sodium dodecyl sulfate-polyacrylamide electrophoresis (SDS-PAGE) method according to Laemmli [31] with Hoefer SE600-IM equipment (Holliston, MA, USA). Acrylamide (99% purity) at 4.5% (*w*/*v*) and 10% (*w*/*v*) were employed for the concentrator and separator gel, respectively. A 70 V initial voltage and a 120 V final voltage were used.

### 2.4. Membrane-Fouling Models

Permeate flux (J) during consecutive filtration stages was calculated according to Equation (8), dividing the permeate volume collected ($V_{p}$) by the membrane area (A) and by the time of sampling (t) [9].

(8) $$J=\frac{V_{p}}{A\;t}$$

Flux decline under constant-pressure filtration for different blocking mechanisms was assessed through the mathematical expressions depicted in Table 1. Dead-end filtration models, formulated by Hermia [17], were systematically derived by Iritani and Katagiri [19]. Crossflow filtration models, presented by Field and Wu [23], were analytically adapted by Wu [24]. Here, v is the permeate volume per unit membrane area, $J_{R}$ is the steady-state flux related to crossflow removal from the surface, $J_{o}$ is the initial permeate flux, t is the filtration time, and Ks, Ki, Kb and Kc are the blocking constants for the standard, intermediate, complete and cake formation mechanisms, respectively. The subscript F refers to crossflow accounting.

The predominant membrane-fouling pattern in each filtration stage was determined based on the best fitting factor (R^2^) for a linear regression analysis. For dead-end models, complete and intermediate blocking mechanisms account for Jvs.t data, while standard blocking and cake formation consider vvs.t data. In the case of the crossflow models, all filtration mechanisms account for J, $J_{o}$, v and t data. Complementarily, if the intermediate blocking mechanism is identified and no cake formation is defined, the maximum permeate volume ($V_{max}$) that should be recollected is estimated according to Equation (9) [19]. This parameter represents the likely irreversible limit when the membrane must be replaced since useful lifetime is reduced [32].

(9) $$V_{max}=\frac{ln(0.4)}{Ki}$$

## 3. Results and Discussion

### 3.1. Laccase Production and Filtration

An overview of the performance in each filtration stage is presented in Table 2. MF is required to reduce bioburden and prevent fouling in UF membranes. For a 0.45 μm pore size membrane, 1.37-fold purification is obtained (16.7 VCF) by means of crossflow MF at constant pressure. This corresponds to 1.13-fold purification (13.3 VCF) reported for *Coriolopsis gallica* laccases [8] and 1.66-fold purification (restricted 10 VCF) achieved for *Pleurotus sajor-caju* laccases [9]. A slight reduction in VCF strongly impacts PF but promotes dead-end MF until the membrane is irreversibly fouled [7]. On the other hand, a VCF rise from 10 to 60 allows for a 3-fold increase in PF, followed by an ACF reduction below one [9]. For the stated VCF, an expected 4.78 ACF was accomplished despite low PF being frequently evidenced throughout MF laccase extracts (ranging from 1.07 to 1.66) [8].

The limited laccase activity (0.166 U/mL) and protein concentration (0.08 mg/mL) in MF permeate leads to less intensive UF treatment. Accordingly, membranes with 1000, 500 and 300 kDa cutoffs reveal deficient performance, with the lowest ACF. In the latest stage, 27% of the enzymatic extract volume has been lost, and the laccase activity in UF 300 permeate is 90.85% similar to that in MF retentate.

UF 100 treatment accomplishes remarkable 6.95-fold purification and 20% recovery. This contrasts with the 10.46-fold purification and 77.4% yield reported for *Cerrena unicolor* laccases [33] under the same filtration conditions referring to temperature and transmembrane pressure. The probable reason is the fixed retentate volume (15 mL), followed by 24.33 VCF compared with 1.03 VCF, which results in low ACF and yield. A PF improvement no longer occurs in subsequent membranes despite the increasing ACF mainly due to the combination of constant pressure and fixed retentate volume (15 mL).

The likely improvement of UF membranes with cutoffs lower than 100 kDa after MF treatment has been demonstrated for *Ganoderma* sp. laccases (10 kDa, 97% recovery) [8], *Coriolopsis gallica* laccases (5 kDa, 86.5% recovery) [7] and *Cerrena unicolor* laccases (10 kDa, 90.6% recovery) (30 kDa, 73.7% recovery) [30,33].

### 3.2. Dynamic Permeate Flux Profile in Constant-Pressure Filtration

**Figure 1:**
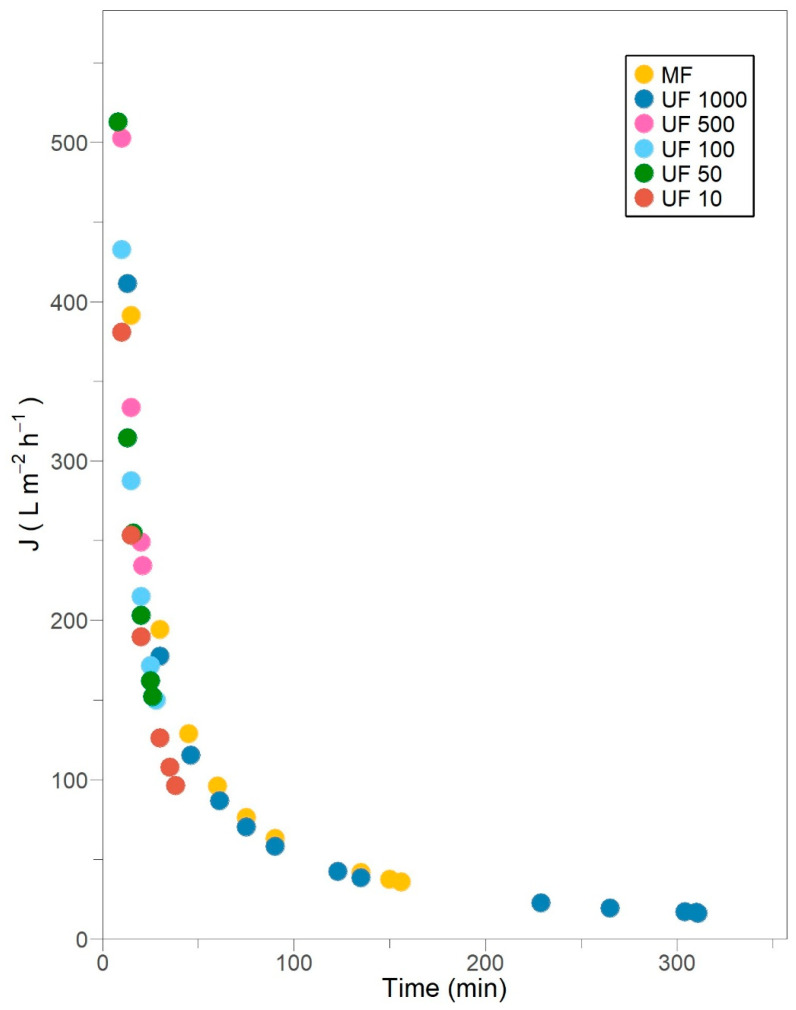
Permeate flux decline in subsequent crossflow filtration stages for *Pleurotus ostreatus* 202 laccase enzymatic extract performed at constant transmembrane pressure (138 kPa) and 18 °C.

The permeate flux decline in subsequent filtration stages is shown in Figure 1. Neither membrane exhibits predominant concentration polarization, which normally takes less than a minute [25], since most of the permeate volume is accounted for after 10 min of operation. Undoubtedly, concentration polarization occurred as an inherent consequence of membrane selectivity related to proteins and enzymes larger than the pore size transported by permeate advection [34,35]. This reversible phenomenon certainly led to an early, quick and notable reduction in permeate flux.

The diffusivity of laccases, as other enzymes like acetylcholinesterase (22 μm^2^/s), F1-ATPase (33 μm^2^/s) and urease (31.8 μm^2^/s) [36], is expected to be exceptionally small, and the mass transfer layer adjacent to the membrane is expected to be thin. Thus, concentration polarization promotes the formation of a flowing layer, but it is not considered a major cause of fouling [34,35]. In this flowing layer, enzymes are assumed to be convectively transported toward the membrane and begin to accumulate due to mechanical rejection [37]. The permeate fluxes of MF and all UF treatments show a sharp decline related to protein and enzyme deposition or adsorption into the pores or on the membrane, which ultimately entails fouling. Most membranes display this behavior for at least 28 min, except the MF and UF 1000 treatments, which certainly could be a consequence of the fixed retentate volume strategy adopted from UF 500 stage onwards.

The MF and UF 1000 stages exhibit a gradual flux decline, tending to a quasi-state-steady flux, where convective deposition is balanced by back transport away from the membrane. This long-term flux loss results in the consolidation of fouling material and the presence of a permanent flow layer [2].

A 153.66 L/m^2^ h permeate flux was reported for *Pleurotus sajor-caju* laccases during crossflow MF (a 0.4 μm pore size and a 0.2 m^2^ permeable area) under 10 VCF and 250 kPa TMP [9]. Such flux is achieved for *Pleurotus ostreatus* 202 laccases after 30 to 45 min of operation, instead of 5 min, under similar VCF but a 0.005 m^2^ permeable area and a 138 kPa TMP. In fact, comparable high purification yields have been observed under a TMP from 34.5 to 172.3 kPa, highlighting the stability of laccases to the shear stress generated within the membrane [7]. Cirillo et al. [37] demonstrated that over extended filtration times, permeation is primarily governed by protein or enzyme deposition, regardless of the applied driving force. On the other hand, an initial 244.4 L/m^2^ h and a final 122.2 L/m^2^ h permeate fluxes have been accomplished for *Cerrena unicolor* laccases during crossflow UF (100 kDa, 0.005 m^2^ permeable area) under 1.03 VCF and 137.9 kPa TMP [33]. For the same membrane characteristics and TMP, an initial 432.8 L/m^2^ h and a final 150 L/m^2^ h permeate fluxes were obtained for *Pleurotus ostreatus* 202 laccases after 28 min but an exceptionally superior 24.33 VCF. Higher permeate flux and retentate concentration were observed at longer residence times, as a result of the formation of a porous layer on the membrane surface [37]. Finally, an average 114 L/m^2^ h permeate flux was estimated for *Ganoderma* sp. laccases during crossflow UF (10 kDa) at 30 °C and 144.7 kPa TMP [7]; meanwhile, an average 192.3 L/m^2^ h permeate flux was calculated for *Pleurotus ostreatus* 202 laccases at 18 °C and similar TMP. Ding et al. [38] stated that permeate flux declines abruptly as the temperature increases due to the expansion of membrane pores and the subsequent propensity for blocking.

In this context, protein–membrane and protein–protein interactions are the leading forces throughout crossflow filtration. Operating conditions related to enzyme or protein concentration, crossflow velocity and TMP, as well as membrane properties accounting for porosity and surface hydrophobicity, become key features for understanding the extent of filtration and the type of fouling occurrence [37].

### 3.3. Analysis of Membrane-Fouling Mechanisms Applied to Crossflow Laccase Filtration

**Figure 2:**
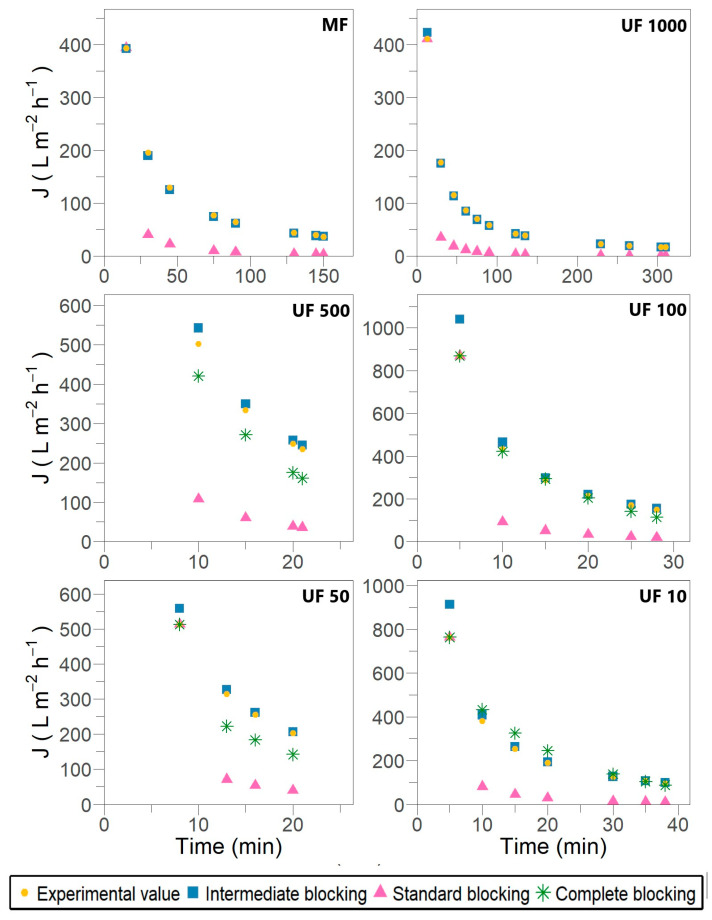
Permeate flux predicted by Hermia’s model for different fouling mechanisms during subsequent crossflow filtration stages for *Pleurotus ostreatus* 202 laccase enzymatic extract performed at constant transmembrane pressure (138 kPa) and 18 °C.

**Figure 3:**
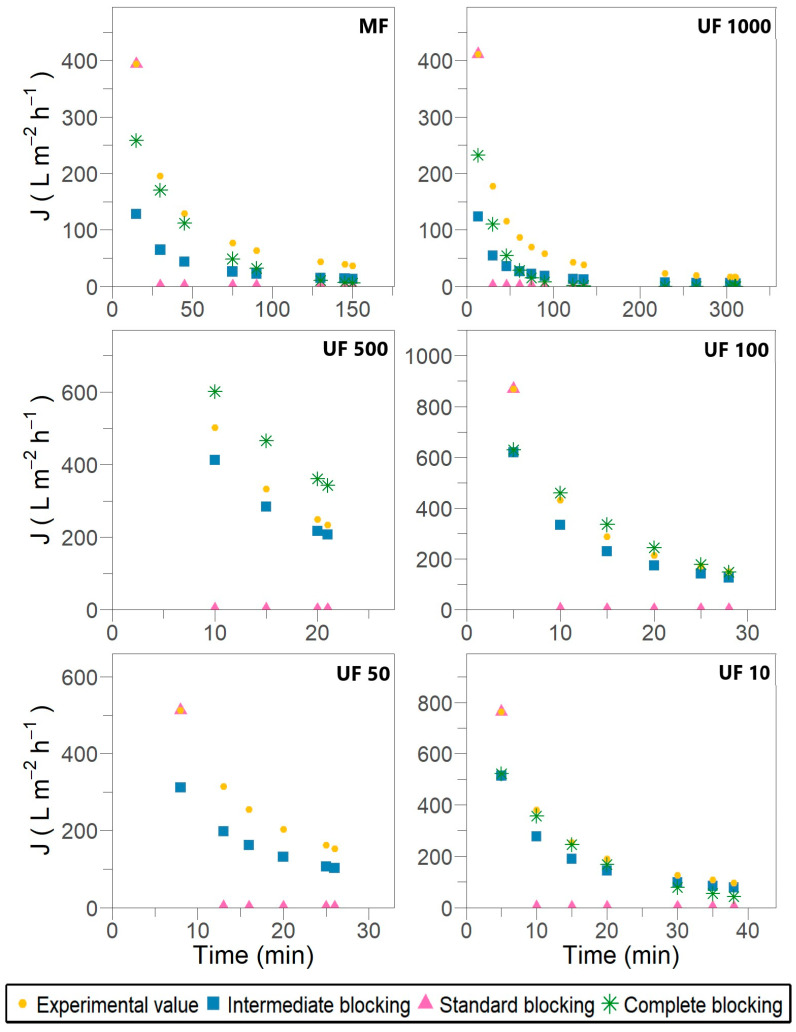
Permeate flux predicted by Field and Wu’s model for different fouling mechanisms accounting for removal during subsequent crossflow filtration stages for *Pleurotus ostreatus* 202 laccase enzymatic extract performed at constant transmembrane pressure (138 kPa) and 18 °C.

Figure 2 and Figure 3 illustrate the fitting of experimental permeate flux to different fouling models described by Hermia [17] for dead-end filtration and modified by Field and Wu [23] for crossflow filtration, respectively.

The outcomes show that membrane fouling in the case of laccase filtration was attributed to pore-blocking mechanisms. No filtration stage reveals cake or gel layer formation over the membrane surface despite the allowance for crossflow removal in the modified models. During the fitting procedure, a recurrent opposite slope inclination was observed compared with the expected one, as defined by the equations in Table 1 and the fitting examples provided by Iritani and Katagiri [19] and Field and Wu [23]. This inconsistency suggests a misinterpretation of the physical meaning underlying the models, despite achieving satisfactory R^2^ values ranging from 0.829 to 0.933. The most likely explanation for this discrepancy could be the fixed retentate volume used in the later stages of the experimental setup.

Fouling in tangential flow filtration is characterized by a disruption of the force balance during the transition from pore blocking to cake filtration, which occurs through the formation of an incompressible cake layer. This transition promotes significant permeation. However, the formation of the incompressible cake is limited by the permeate volume, which may lead to a loss in membrane permeability of approximately 40% to 60% [39]. Furthermore, the filtration behavior of laccases, as with other enzymes such as lysozymes, differs from that of proteins like BSA and pepsin, as fouling is primarily caused by pore blockage and constriction rather than by the formation of a dense cake layer [6].

The results shown in Figure 2 and Figure 3 suggest that individual blocking models are unable to fully describe the flux decline throughout the entire filtration process. As previously reported by Bowen et al. [40] and subsequently validated by Lay et al. [6], the most severe membrane fouling during protein crossflow filtration can be characterized by a combination of successive and simultaneous fouling mechanisms, starting with complete blocking, followed by standard blocking, and concluding with intermediate blocking. However, during different stages of laccase filtration, an alternating pattern of intermediate and complete pore blocking was observed in the early and mid-filtration phases. In contrast, the standard blocking mechanism typically appeared during the late-stage filtration and, in some cases, was entirely absent.

#### 3.3.1. Complete Pore Blocking with Allowance for Crossflow Removal (*n = 2*)

This fouling mechanism considers the accumulation of molecules on the membrane surface where a constant probability of blocking an open pore during filtration is accounted for [19,23]. Transmembrane pressure and membrane surface porosity are the main influencing factors [20]. PES membranes are characterized by an asymmetric structure, consisting of a thin, dense top layer with fine pores, and an internal sponge-like, heterogeneous network with higher porosity [37]. This sponge-like microstructure makes PES membranes more susceptible to pore fouling due to their highly porous internal structure [2,41]. On the other hand, the use of a higher TMP was found to exacerbate enzyme deposition, as internal deposits connect with those on the membrane surface, forming complex structures that completely clog the pores [39].

Despite Hermia’s model demonstrating elevated R^2^ values across the filtration stages presented in Table 3, the fitting adjustment with experimental data is unsatisfactory, as observed in Figure 2. No complete pore blocking is evidenced for the MF and UF 1000 stages, which does not align with the principle of the mechanism and membrane performance. In the UF 100 stage, an R^2^ of 0.947 is determined, where the calculated flux overlaps with the experimental value. In contrast, in the UF 50 stage, the calculated flux is notably lower than the experimental one, despite an R^2^ of 0.973 being reported. In Field and Wu’s model, the parameter R^2^ results appropriate for ruling out options because, in the UF 50 stage, an R^2^ of 0.503 effectively assesses no adjustment. Therefore, the lack of sensitivity in dead-end models leads to an inadequate description of the complete pore-blocking mechanism in laccase filtration.

The MF, UF 1000, UF 100 and UF 10 filtration stages are the most affected by complete pore blockage, as shown in Figure 3. This results in the near-total occlusion of certain pore openings by enzyme deposits. The filtration constant Kb_F_ represents the membrane surface area blocked per unit of permeate volume and per unit of initial membrane porosity [19,21]. When crossflow removal is considered, a clear pattern of behavior for Kb_F_ emerges in the affected stages, as presented in Table 3. As membrane pore size decreases, Kb_F_ increases, indicating accelerated fouling and a shorter filtration time before significant flux decline. This trend is further supported by the value of J_R_, which represents the steady-state flux under the assumption of a single fouling mechanism [23]. The J_R_ values suggest that in the initial stages (MF and UF 1000), the first 60 min is dominated by complete pore blocking, whereas in UF 100 and UF 10, this mechanism is only relevant during the first 15 to 20 min.

#### 3.3.2. Intermediate Pore Blocking with Allowance for Crossflow Removal (*n = 1*)

As membrane pores are already occluded near their entry points, this fouling mechanism postulates that certain enzymes may be deposited on other enzymes previously settled [16]. According to the dead-end filtration model, the elevated R^2^ value in Table 4 indicates that the intermediate blocking mechanism is present throughout all filtration stages. The calculated flux aligns closely with the experimental values shown in Figure 2. However, when considering crossflow removal, the calculated flux in all filtration stages is lower than the experimental flux, as illustrated in Figure 3. The UF 500, UF 100 and UF 10 stages are the most affected by intermediate blocking.

The initial protein–membrane interactions in diminutive pores (approximately 5 nm) and the terminal protein–protein interactions are pivotal to the adsorption phenomenon inside the pores and on the membrane wall [2]. The crossflow constant Ki_F_ has the same significance as Kb_F_; therefore, its increase enhances fouling over filtration time. In particular, in UF 100 and UF 10—previously affected by complete blocking—Ki_F_ is higher than Kb_F_, indicating a significant combined effect. This behavior is supported by the SDS-PAGE analysis (Supplementary Material Figure S1) of the UF 100 retentate, in which 67, 62 and 59 kDa laccases were identified. Once again, the satisfactory agreement of Field and Wu’s model has been confirmed. In accordance with the J_R_ values, the intermediate blocking mechanism predominates up to 10 min in UF 500 and from 15 to 20 min in UF 100 and UF 10, as observed in Figure 3.

#### 3.3.3. Pore Filling or Conventional Standard Pore Blocking (*n = 1.5*)

Pore filling is caused by the adsorption of molecules on the pore wall, which is characterized by the periodic blocking and unblocking of the pores due to the coupling of membrane straight pores with enzymatic foulants [6,21]. Hydrophobic membranes such as PES promote nonspecific adsorption and even cause structural changes in proteins, resulting in a rapid flux decline [2,41]. The calculated flux related to standard blocking is significantly lower than the experimental flux; therefore, the correlation with the experimental data is notably inaccurate (Figure 2 and Figure 3), despite the high R^2^ values observed in both models (Table 5). The standard blocking mechanism could not become dominant in laccase filtration because most membranes exhibit complete and partial pore blocking, as revealed in Section 3.3.1 and Section 3.3.2.

Only the MF and UF 1000 stages were partially affected by standard blocking up to 100 min, as shown in Figure 3. Cirillo et al. [37] demonstrated that wider pores of 0.45 µm and larger membranes tend to be restricted by protein deposits accumulating on their walls. Moreover, *P. ostreatus* secretes a wide variety of laccase isoforms during fermentation. Díaz and Díaz-Godínez [42] detected four extracellular isoforms with molecular weights of 29, 38, 47 and 65 kDa in *P. ostreatus* ATCC 32783 related to pH influence. For the same fungus strain, Armas-Tizapantzi et al. [43] identified three intracellular isoforms with molecular weights of 34, 37 and 50 kDa associated with the addition of copper to fermentation media.

As fouling is caused by internal pore blocking, it becomes independent of the crossflow velocity and is not mediated by back-diffusion from the membrane surface; therefore, no limiting value for the permeate flux is attained (J_R_ → 0) [23]. Standard pore-blocking models postulate that the pore volume decreases proportionally to the filtrate volume per unit of membrane area. Consequently, it is hypothesized that Ks and Ks_F_ have equivalent magnitudes and demonstrate a positive correlation with the severity of membrane fouling [19,21]. Consistently, in Field and Wu’s model, the higher Ks_F_ values and lower J_R_ (Table 5) are obtained in the MF and UF 1000 stages.

### 3.4. Degree of Membrane Fouling

Intermediate pore blocking hinders the progression of standard pore blocking due to the prevention of further shrinkage once a pore on the surface becomes occluded [44]. In the absence of cake formation at any filtration stage, the available membrane area is known to decrease in proportion to the deposition of particles over time due to intermediate blocking. This results in a rearrangement of the membrane area as a function of time [16]. As Ki increases, the available membrane area experiences a substantial decrease due to the accumulation of irreversible foulants, and a permeate flux reduction will be achieved [16,44]. According to Slimane et al. [45], a permeate flux reduction of approximately 30% was demonstrated, although a 15s backwash was performed for UF membranes every 90 min. Correspondingly, a 45% permeate flux reduction was documented for activated sludge suspension, despite the implementation of a backwash cycle in UF membranes with combined standard and intermediate pore-blocking fouling mechanisms [44].

**Figure 4:**
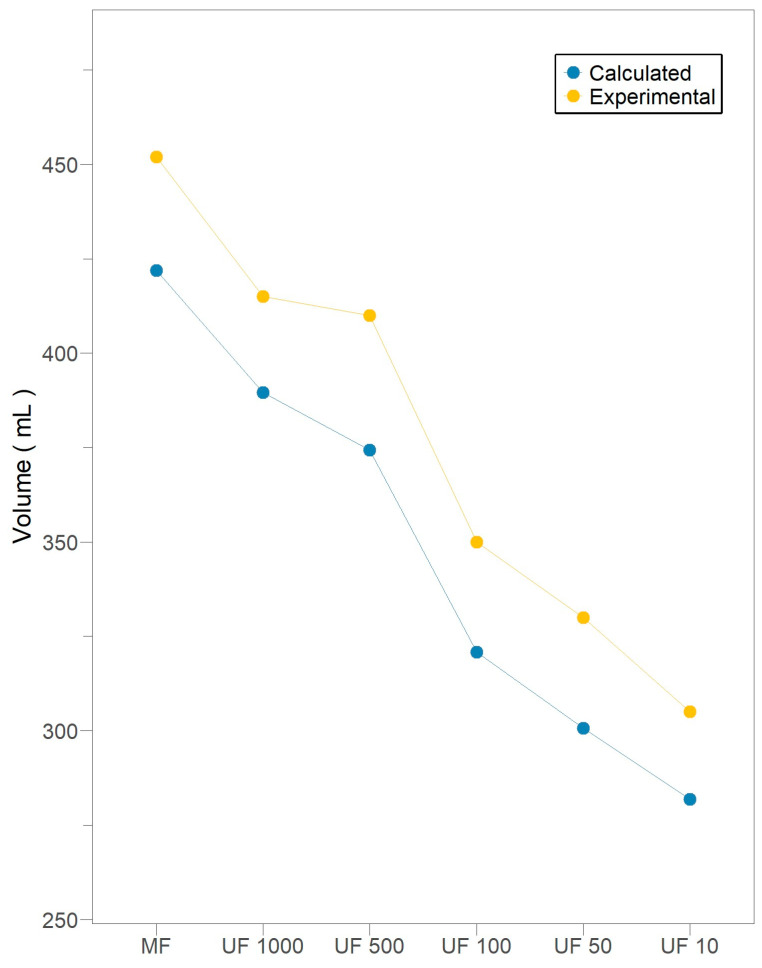
Total permeate volume during subsequent crossflow filtration stages for *Pleurotus ostreatus* 202 laccase enzymatic extract performed at constant transmembrane pressure (138 kPa) and 18 °C.

In this context, Figure 4 illustrates the permeate volume for a 40% flux reduction at each filtration stage, where it is hypothesized that the accumulation of irreversible foulants becomes significant mainly due to the impact of the intermediate pore-blocking mechanism. In all filtration stages, there is evidence of moderate, non-exhaustive use of the membranes, where the permeate volume collected is approximately 10% greater than the calculated critical value. Therefore, no membrane was subjected to a fouling process that could not be reversed by backwashing.

### 3.5. Conclusions

The performance of constant-pressure filtration indicates that crossflow MF aims to remove impurities (cells and debris) in the initial extract, resulting in reduced protein concentrations and laccase activity in the permeate. The low ACF in the following stages (UF 1000, UF 500 and UF 300) reflects its lack of contribution to laccase concentration. Indeed, crossflow UF 100 results in the highest purification factor, and subsequent membranes cannot improve it despite the ACF improvement. The analysis of the dynamic flux profile reveals that no filtration stage demonstrates a significant occurrence of concentration polarization. This is due to the low diffusivity characteristic of enzymes, which results in a thin layer adjacent to the membrane. Only the MF and UF 1000 stages experience convective deposition, as the flux tends to a steady state, resulting in the likely consolidation of fouling. However, the main cause of fouling is deposition on the pores and adsorption into the pores, which are present in all filtration stages. Therefore, the pore-blocking mechanism at each filtration stage is satisfactorily assessed by Field and Wu’s models compared with the originally formulated Hermia’s model for dead-end filtration. When crossflow removal is considered, the fitting parameter R^2^ is appropriate for eliminating options.

Initially, the membrane is flooded by the complete blockage of the pores. The MF and UF 1000 stages most deeply present this fouling mechanism during the first 60 min, while the UF 100 and UF 10 stages do so around 15 min. The filtration constant Kb_F_ gradually increases from 4.64 × 10^−4^ to 12.60 × 10^−4^ (s^−1^). Subsequently, despite the presence of intermediate pore blocking in all filtration stages, UF 100 demonstrates a notable effect. The latter finding is corroborated by the presence of 67, 62, and 59 kDa laccases in the retentate.

The absence of cake formation guarantees a proportional decrease in available membrane area, attributable to the deposition of particles during intermediate blocking. Consequently, the permeate volume related to a 40% flux reduction emerges as a reliable indicator of fouling severity. Thus, all filtration stages exhibit moderate, non-exhaustive use of membranes, as the permeate volume is around 10% greater than the calculated critical value.

## Figures and Tables

**Table 1:** Fitting equations for individual pore-blocking mechanisms under constant-pressure filtration.

Pore-Blocking Mechanism	Dead-End Model	Crossflow Model
Standard blocking	$\frac{t}{v}=\frac{Ks}{2}t+\frac{1}{J_{o}}$	$\frac{J_{o}^{0.5}-J^{0.5}}{t}=\frac{{Ks}_{F}\;v}{t}-{Ks}_{F}\;J_{R}$
Intermediate blocking	$\frac{1}{J}=Ki\;t+\frac{1}{J_{o}}$	$\frac{ln\left(J_{o}/J\right)}{t}=\frac{{Ki}_{F}\;v}{t}-{Ki}_{F}\;J_{R}$
Complete blocking	$\ln\left(J\right)=-Kb\;t+ln(J_{o})$	$\frac{J_{o}-J}{t}=\frac{{Kb}_{F}\;v}{t}-{Kb}_{F}\;J_{R}$
Cake formation	$\frac{t}{v}=\frac{Kc}{2}v+\frac{1}{J_{o}}$	$\frac{\left(1/J-1/J_{o}\right)}{t}=\frac{{Kc}_{F}\;v}{t}-K_{cF}\;J_{R}$

**Table 2:** Results for sequential crossflow filtration conducted on *Pleurotus ostreatus* 202 laccase enzymatic extract.

Stage	V (mL)	Laccase (U/mL)	Protein (mg/mL)	SA (U/mg)	VCF	ACF	R (%)	PF
**MF**					16.67	4.78	29	1.37
Initial extract	500	0.217	0.08	2.70				
Retentate	30	1.038	0.28	3.70				
**UF 1000**					10.44	2.04	20	1.49
MF permeate	470	0.166	0.08	2.10				
Retentate	45	0.339	0.11	3.10				
**UF 500**					28.33	0.80	3	1.79
UF 1000 permeate	425	0.122	0.09	1.40				
Retentate	15	0.097	0.04	2.40				
**UF 300**					26.67	0.89	3	0.89
UF 500 permeate	400	0.114	0.07	1.60				
Retentate	15	0.101	0.07	1.40				
**UF 100**					24.33	4.97	20	6.95
UF 300 permeate	365	0.095	0.07	1.40				
Retentate	15	0.473	0.05	9.50				
**UF 50**					23.00	5.33	23	3.20
UF 100 permeate	345	0.071	0.06	1.20				
Retentate	15	0.379	0.10	3.80				
**UF 10**					21.33	5.65	26	4.04
UF 50 permeate	320	0.046	0.05	0.90				
Retentate	15	0.260	0.07	3.70				

**Table 3:** Adjusted parameters for dead-end and crossflow filtration models under complete pore-blocking fouling mechanism.

Stage	Hermia’s Model		Field and Wu’s Model		
	R^2^	Kb × 10^4^ (m^−1^)	R^2^	Kb_F_ × 10^4^ (m^−1^)	J_R_ (L/m^2^ h)
MF	0.922	2.58	0.939	4.64	59.39
UF 1000	0.864	1.38	0.954	7.31	20.76
UF 500	0.965	14.60	0.948	8.51	506.45
UF 100	0.947	12.07	0.933	10.51	283.20
UF 50	0.973	10.70	0.503	1.70	1248.08
UF 10	0.932	9.47	0.932	12.60	157.19

**Table 4:** Adjusted parameters for dead-end and crossflow filtration models under intermediate pore-blocking fouling mechanism.

Stage	Hermia’s Model		Field and Wu’s Model		
	R^2^	Ki (m^−1^)	R^2^	Ki_F_ (m^−1^)	J_R_ (L/m^2^ h)
MF	0.998	0.181	0.996	30.60	32.35
UF 1000	0.999	0.196	0.989	36.50	18.74
UF 500	0.999	0.204	0.979	13.50	452.00
UF 100	0.999	0.238	0.972	16.70	325.87
UF 50	0.999	0.254	0.989	22.40	197.95
UF 10	0.999	0.271	0.962	20.30	230.84

**Table 5:** Adjusted parameters for dead-end and crossflow filtration models under pore filling mechanism.

Stage	Hermia’s Model		Field and Wu’s Model		
	R^2^	Ks (m^−1^)	R^2^	Ks_F_ × 10^2^ (s^−0.5^ m^−0.5^)	J_R_ (L/m^2^ h)
MF	0.998	21.72	0.998	29.83	0.0049
UF 1000	0.999	23.56	0.996	31.17	0.0034
UF 500	0.999	24.46	0.993	21.21	0.0507
UF 100	0.999	28.54	0.991	23.61	0.0362
UF 50	0.999	30.42	0.997	28.18	0.0212
UF 10	0.999	32.48	0.988	26.09	0.0253

## Data Availability

The raw data supporting the conclusions of this article will be made available by the authors upon request.

## Supplementary Materials

The following supporting information can be downloaded at: https://www.mdpi.com/article/10.3390/membranes15080226/s1, Figure S1: SDS-PAGE of UF 100 retentate. Lane M—protein standards. Lane UF 100—crude extract in retentate.

## Abbreviations

The following abbreviations are used in this manuscript:

${Lac}_{r}$	Laccase activity in retentate
${Lac}_{o}$	Laccase activity in feed
$V_{r}$	Retentate volume
$V_{o}$	Initial extract volume
${SA}_{r}$	Specific activity of retentate
${SA}_{o}$	Specific activity of feed
J	Permeate flux at time t
$V_{p}$	Permeate volume collected
A	Membrane area
t	Time of sampling
v	Permeate volume per unit membrane area
$J_{O}$	Initial permeate volume
$J_{R}$	Steady-state flux related to crossflow removal from surface
Ks	Blocking constant for standard dead-end filtration mechanism
${Ks}_{F}$	Blocking constant for standard crossflow filtration mechanism
Ki	Blocking constant for intermediate dead-end filtration mechanism
${Ki}_{F}$	Blocking constant for intermediate crossflow filtration mechanism
Kb	Blocking constant for complete dead-end filtration mechanism
${Kb}_{F}$	Blocking constant for complete crossflow filtration mechanism
Kc	Blocking constant for cake formation dead-end filtration mechanism
${Kc}_{F}$	Blocking constant for cake formation crossflow filtration mechanism
$V_{max}$	Maximum permeate volume
