# Supplementary figures and images for: A Model-Driven Approach to Assessing the Fouling Mechanism in the Crossflow Filtration of Laccase Extract from Pleurotus ostreatus 202
Source: Membranes (Basel). 2025 Jul 29;15(8):226. doi: 10.3390/membranes15080226 (PMC12388754; doi:10.3390/membranes15080226)

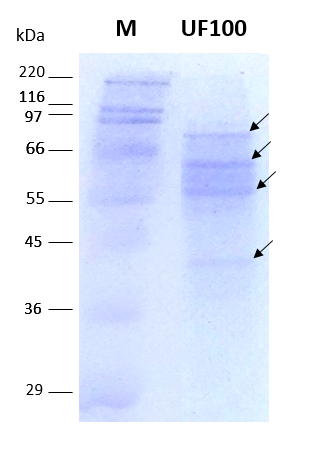

Supplement: Supplementary file 1 [file membranes-15-00226-s001.zip › membranes-3696157-supplementary/membranes-3696157-supplementary.png]
